# Asymmetry of cerebral glucose metabolism in very low-birth-weight infants without structural abnormalities

**DOI:** 10.1371/journal.pone.0186976

**Published:** 2017-11-02

**Authors:** Jae Hyun Park, Chun Soo Kim, Kyoung Sook Won, Jungsu S. Oh, Jae Seung Kim, Hae Won Kim

**Affiliations:** 1 Department of Pediatrics, Keimyung University Dongsan Medical Center, Daegu, Republic of Korea; 2 Department of Nuclear Medicine, Keimyung University Dongsan Medical Center, Daegu, Republic of Korea; 3 Department of Nuclear Medicine, Asan Medical Center, University of Ulsan College of Medicine, Seoul, Korea; Banner Alzheimer's Institute, UNITED STATES

## Abstract

Even when structural abnormalities are not observed on the brain magnetic resonance images (MRI) of very low-birth-weight (VLBW) infants, such infants are at increased risk for poor neurodevelopment. The aim of the present study was to evaluate cerebral glucose metabolism in VLBW infants without apparent structural abnormalities on MRI.

**Methods:**

Thirty-six VLBW infants who underwent F-18 fluorodeoxyglucose (F-18 FDG) brain PET and MRI were prospectively enrolled, while infants with evidence of parenchymal brain injury on MRI were excluded. The regional glucose metabolic ratio and asymmetry index were calculated. The asymmetry index more than 10% (right > left asymmetry) or less than -10% (left > right asymmetry) were defined as abnormal. Regional cerebral glucose metabolism were compared between right and left cerebral hemispheres, and between the following subgroups: multiple gestations, premature rupture of membrane, bronchopulmonary dysplasia, and low-grade intraventricular hemorrhage.

**Results:**

In the individual analysis, 21 (58.3%) of 36 VLBW infants exhibited asymmetric cerebral glucose metabolism. Fifteen infants (41.7%) exhibited right > left asymmetry, while six (16.7%) exhibited left > right asymmetry. In the regional analysis, right > left asymmetry was more extensive than left > right asymmetry. The metabolic ratio in the right frontal, temporal, and occipital cortices and right thalamus were significantly higher than those in the corresponding left regions. In the subgroup analyses, the cerebral glucose metabolism in infants with multiple gestations, premature rupture of membrane, bronchopulmonary dysplasia, or low-grade intraventricular hemorrhage were significantly lower than those in infants without these.

**Conclusion:**

VLBW infants without structural abnormalities have asymmetry of cerebral glucose metabolism. Decreased cerebral glucose metabolism are noted in infants with neurodevelopmental risk factors. F-18 FDG PET could show microstructural abnormalities not detected by MRI in VLBW infants.

## Introduction

Over the last decade, improvements in perinatal care have resulted in increased rates of survival for very low-birth-weight (VLBW) infants [1, 2]. However, an increase in preterm births and remarkable improvements in survival for infants have outpaced any concomitant decrease in rates of long-term neurodevelopmental disability [3]. As many as 42–47% of cerebral palsy cases in children can be attributed to preterm births [4, 5]. In addition, subtle motor and cognitive dysfunction due to high-grade intraventricular hemorrhage (IVH) or white matter injury has been associated with extreme prematurity, most often resulting in regulatory, attentional, or adaptive impairments [6]. Furthermore, VLBW infants without severe brain injury are at risk for cognitive dysfunction and may require more support than do full-term infants.

Brain magnetic resonance imaging (MRI) is considered the most sensitive and specific modality for diagnosing brain injury and predicting neurodevelopmental outcomes in VLBW infants. The most extensively studied MRI abnormalities shown to predict neurodevelopmental outcomes are white matter lesions (e.g., periventricular leukomalacia [PVL], punctate white matter lesions, and diffuse excessive high signal intensities) [7]. However, although conventional MRI can be used to detect macrostructural brain abnormalities in VLBW infants, such images are unable to detect micropathology in VLBW infants [8]. Indeed, previous studies have demonstrated that some children with cerebral palsy or cognitive dysfunction exhibit no apparent abnormalities on conventional MRI [9, 10].

However, F-18 fluorodeoxyglucose (F-18 FDG) positron emission tomography (PET) imaging may be helpful in detecting cerebral glucose metabolic abnormalities at a much earlier stage, prior to the development of structural and morphologic abnormalities [11]. Previous studies have demonstrated that patterns of cerebral glucose metabolism are correlated with the later development of different types of cerebral palsy in children [12, 13]. Although there have been no reports regarding cerebral glucose metabolism in VLBW infants, it is generally accepted that many neurodegenerative diseases are associated with significantly decreased glucose metabolism on F-18 FDG) PET images, even though conventional MRI images reveal no specific abnormalities [14, 15]. Thus, the aim of the present study was to evaluate cerebral glucose metabolism in VLBW infants without structural abnormalities on brain MRI.

## Material and methods

### Subjects

VLBW infants (<1500 g) born at less than 30 weeks gestation admitted to the neonatal intensive care unit of Keimyung University Dongsan Medical Center between December 2014 and October 2015 were included in the present prospective study. All infants underwent MRI scans at term-equivalent age, and infants who exhibited evidence of parenchymal brain injury on MRI (cystic PVL, congenital cerebral anomaly, or grade 3 or 4 IVH) were excluded [16]. In all infants, F-18 FDG PET was performed at term-equivalent age. The institutional review board of Dongsan Medical Center approved the present study, and written informed consent was obtained from the parents/guardians of all participants.

### Protocol for ^18^F-FDG PET acquisition

A PET/CT system was used to acquire F-18 FDG PET images (Biograph mCT-64, Siemens Healthcare, Knoxville, TN). Infants received intravenous administration of 3.7 MBq/kg. Each infant was sedated using thiopental 40 minutes after F-18 FDG injection, and images were acquired 50 minutes after injection. A non-enhanced low-dose CT scan was obtained for attenuation correction and localization. A light, foam-rubber holder was used for fixation of the head. The PET images were reconstructed iteratively using ordered subset expectation maximization. Attenuation correction of PET images was performed using attenuation data from CT images. All fusion images were viewed using dedicated workstations for each PET/CT system.

### Quantitative analyses

Image processing was performed using SPM12 (Wellcome Department of Imaging Neuroscience, Institute of Neurology, University College London) within MATLAB 2013a (MathWorks Inc., MA, USA) and MRIcro version 1.37 (Chris Rorden, Columbia, SC, USA, www.mricro.com). Quantitative analyses were conducted on volumes of interest (VOIs) using the software program PMOD (PMOD Technologies Ltd, Zurich, Switzerland), as previously described [17]. All reconstructed PET images were co-registered to the corresponding MR images and spatially normalized to a neonate brain atlas using a neonate brain MRI template [18]. For analysis of cerebral glucose metabolism, 24 regional VOIs were identified via the neonate automated anatomic labeling (nAAL) template, as previously described [18, 19]. We investigated the following regional VOIs: the central region; lateral, medial, and orbitofrontal lobes; lateral and medial temporal lobe; lateral parietal lobe; lateral and medial occipital lobe; caudate nucleus; putamen; and thalamus. The VOI template was automatically applied directly to the spatially normalized individual PET images for the analysis of cerebral glucose metabolism.

The activity concentration was calculated for each VOI of the F-18 FDG PET images. The activity concentration in the whole brain was also calculated as a reference. The regional cerebral glucose metabolic ratio (CMRgl) for each VOI was calculated as follows: CMRgl = count of regional VOI / count of whole brain. The asymmetry index (AI_voi_) for each VOI was calculated as follows: AI_voi_ = (count of right VOI—count of left VOI) / (count of right VOI + count of left VOI) × 200. In the regional analysis, AI_voi_ values more than 10% (right > left asymmetry) or less than -10% (left > right asymmetry) were defined as abnormal [20]. The asymmetry of cerebral glucose metabolism was also evaluated using voxel based morphometry (VBM): We created mirror images of each PET image, following which we spatially normalized both the original PET and mirror images. Overlay masks were generated from the averaged spatially normalized PET image using a region-growing algorithm, as previously described [21]. The voxelwise asymmetry index (AI_vbm_) was calculated using the following formula: AI_vbm_ = (∑X_*orig*_ − (∑X_*mirror*_) ⁄(∑X_*orig*_ + ∑X_*mirror*_) × 200, where *orig* and *mirror* represent the original PET and mirror images, respectively, and *X* denotes voxel intensity of the PET image. The mean values of AI_vbm_ were overlaid onto a standardized neonate brain MRI.

In the individual analysis, infants with abnormal AI_voi_ values in any regional VOI were classified into the abnormal AI_voi_ group. We also compared regional CMRgl between VLBW infants with and without risk factors for poor neurodevelopmental outcome, including premature rupture of membrane (PROM), bronchopulmonary dysplasia (BPD), multiple gestations (MG), grade 1 or 2 IVH.

### Statistical analyses

The regional CMRgl values were compared between the left and right cerebral hemispheres using paired samples t-tests. The regional CMRgl values were compared between each subgroup (MG, PROM, BPD, and IVH) using two independent t-tests. The *P* values were corrected for multiple comparisons via false discovery rate correction. One-sample t-tests were used to determine whether the AI_voi_ of each region was higher than 0. A *P* value of less than 0.05 was considered to indicate statistical significance. Data for all study variables are expressed as the mean ± SD.

## Results

### Characteristics

**Fig 1:**
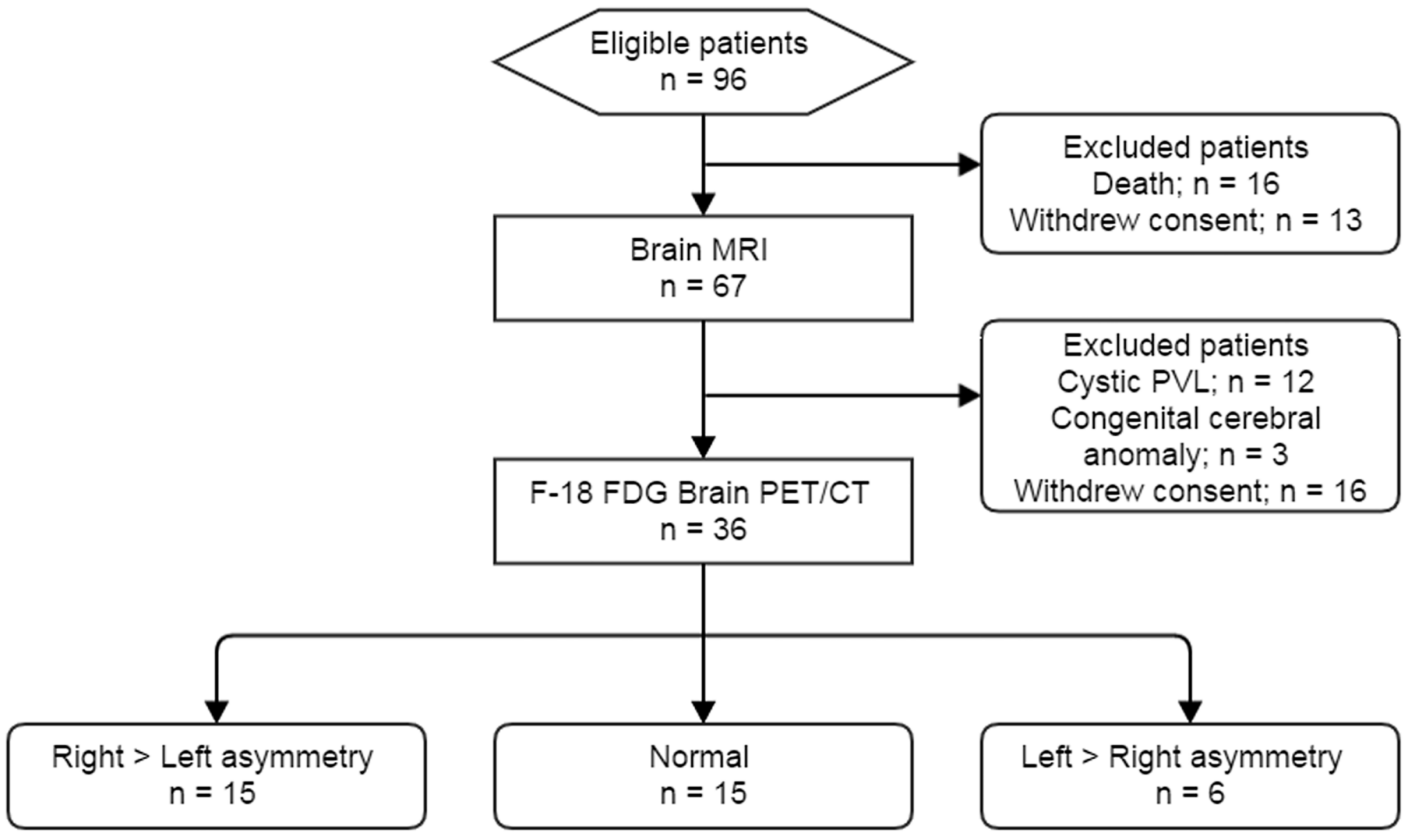
Flow diagram of the study population. PVL: periventricular leukomalacia.

Of the 96 live-born VLBW infants eligible for participation, 80 (83.3%) survived to term-equivalent age. Among these, the parents of 29 infants refused to participate in the study, and 15 further infants were excluded due to evidence of parenchymal brain lesions on MRI: cystic PVL (n = 12) and congenital cerebral anomaly (n = 3). Therefore, the final sample consisted of 36 VLBW infants who underwent F-18 FDG brain PET (Fig 1). The mean GA was 27.2 ± 1.3 weeks, and mean birth weight was 925.0 g. The mean chronological age at evaluation of F-18 FDG PET was 10.0 weeks. No significant differences in gestational age, birth weight, or sex were observed among the PROM, BPD, MG, and IVH subgroups. Table 1 summarizes the clinical characteristics of the infants.

**Table 1:** Characteristics of very-low-birth-weight infants.

Characteristics	Infant data
Mean gestational age at birth (wk)	27.2 ± 1.3
Mean birth weight (g)	925.0 ± 207.2
Chronological age at F-18 FDG PET (wk)	10.0 ± 2.2
Male sex, no. (%)	14 (38.9)
Premature rupture of membrane, no. (%)	14 (38.9)
Bronchopulmonary dysplasia, no. (%)	8 (22.2)
Multiple gestation, no. (%)	13 (36.1)
Apgar score	
at 1 min, mean (SD)	5.0 ± 1.4
at 5 min, mean (SD)	7.3 ± 1.3

### Cerebral glucose metabolism

**Fig 2:**
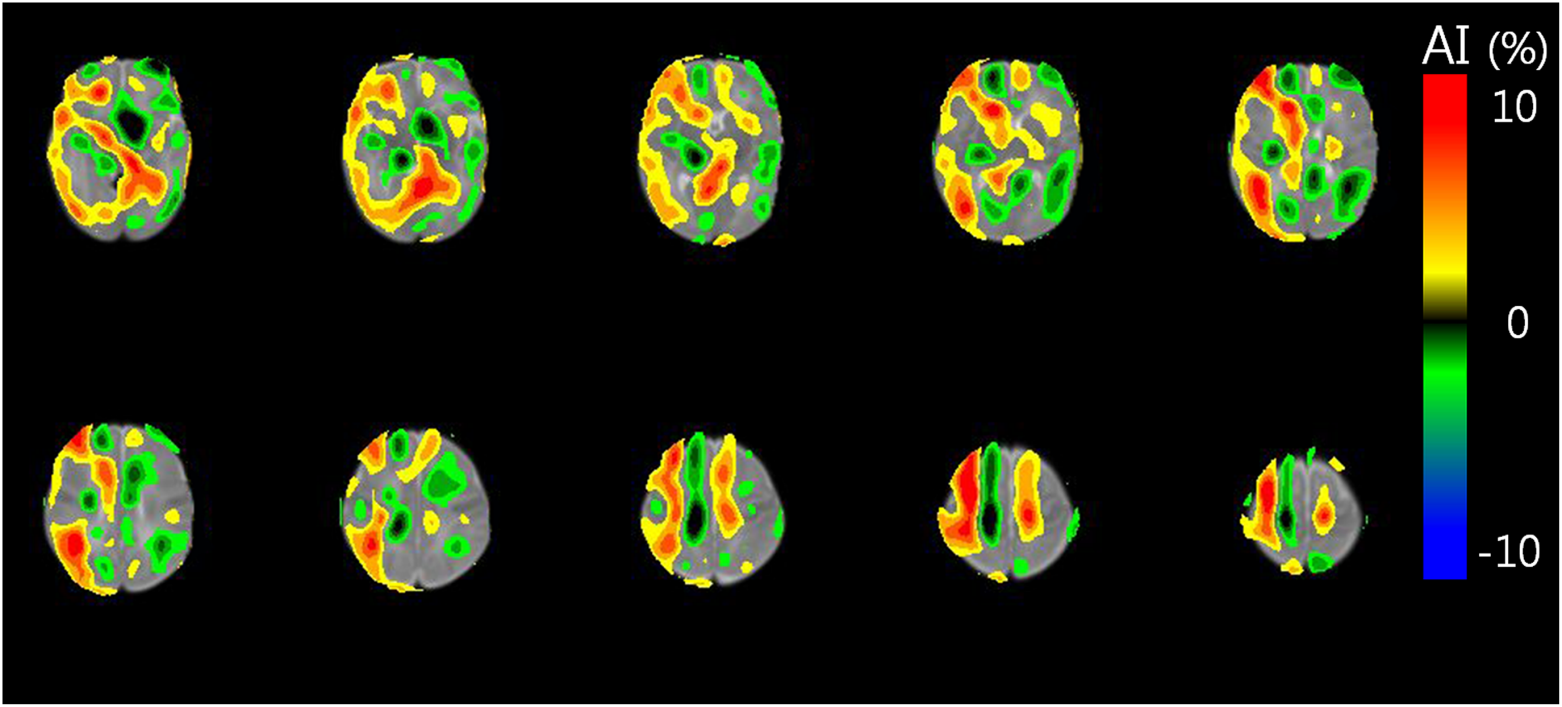
Voxelwise asymmetry index (AI_vbm_) of cerebral glucose metabolism in a very low-birth-weight infant. Voxel-based morphometry revealed right dominant asymmetry of cerebral glucose metabolism. Red indicates a region of relatively high glucose metabolism (where AI_vbm_ is positive) in comparison to corresponding regions. Blue indicates a region of relatively low glucose metabolism (where AI_vbm_ is negative) in comparison to corresponding regions.

The CMRgl of the right medial and orbitofrontal, right lateral temporal, right lateral and medial occipital lobes, and right thalamus were significantly higher than those of the corresponding regions in the left hemisphere (*P* <0.001, *P* <0.001, *P* = 0.002, *P* = 0.002, *P* = 0.008, and *P* <0.001, respectively). The CMRgl of the right putamen was significantly lower than that of the left putamen (*P* = 0.019). Table 2 shows the mean AI_voi_ of each VOI in VLBW infants. The VBM analysis also revealed right dominant asymmetry of cerebral glucose metabolism. The mean AI_vbm_ values in the right cerebral hemisphere were positive, while the mean AI_vbm_ values in the left cerebral hemisphere were negative (Fig 2).

**Table 2:** Cerebral metabolic ratio and asymmetry index in very low-birth-weight infants.

VOI*	Cerebral metabolic ratio		Asymmetry index (%)	*P* value^†^
	Left	Right		
Central region	1.06 ± 0.05	1.07 ± 0.04	0.6 ± 5.0	0.500
Lateral frontal lobe	0.90 ± 0.04	0.90 ± 0.05	-0.6 ± 4.2	0.477
Medial frontal lobe	0.89 ± 0.05	0.92 ± 0.06	3.8 ± 4.9	< 0.001^‡^
Orbital frontal lobe	0.89 ± 0.04	0.91 ± 0.04	2.0 ± 2.1	< 0.001^‡^
Lateral temporal lobe	1.03 ± 0.05	1.06 ± 0.03	2.9 ± 4.5	0.002^‡^
Medial temporal lobe	1.19 ± 0.12	1.20 ± 0.12	2.3 ± 3.1	0.231
Lateral parietal lobe	0.87 ± 0.04	0.86 ± 0.04	-1.60 ± 3.2	0.06
Lateral occipital lobe	0.87 ± 0.05	0.89 ± 0.04	3.0 ± 4.7	0.002^‡^
Medial occipital lobe	0.96 ± 0.04	0.98 ± 0.03	1.4 ± 2.8	0.008^‡^
Caudate nucleus	0.89 ± 0.09	0.90 ± 0.09	1.0 ± 7.2	0.477
Putamen	1.35 ± 0.12	1.32 ± 0.11	-2.6 ± 5.8	0.019^‡^
Thalamus	1.35 ± 0.19	1.42 ± 0.18	5.3 ± 7.5	< 0.001^‡^

*VOI: volume of interest.

^†^Comparison of cerebral metabolic ratios between right and left hemispheres.

^‡^Statistically significant results.

**Fig 3:**
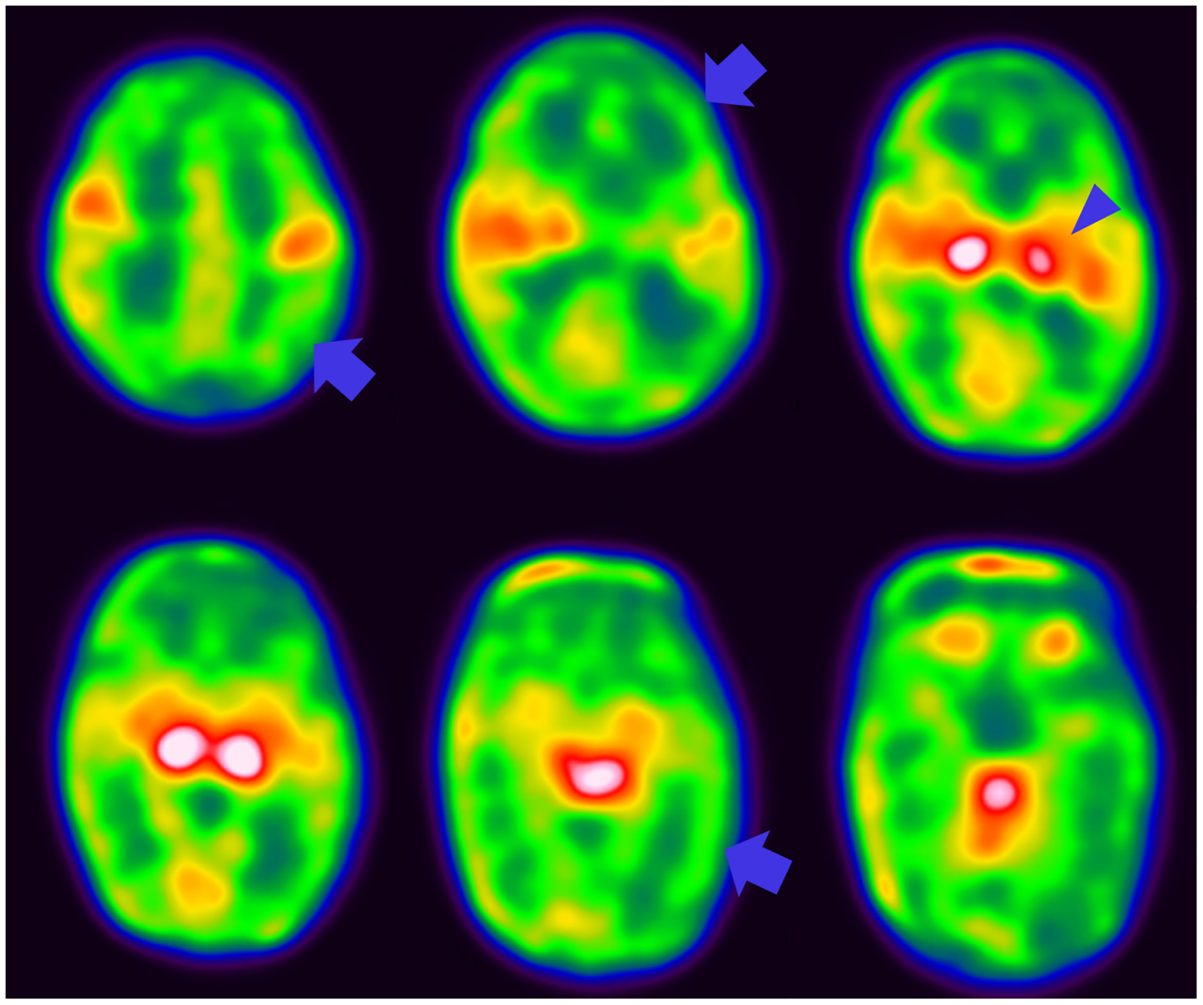
A representative F-18 FDG brain PET image from a very low-birth-weight infant. It shows relatively low glucose metabolism in the right cerebral cortex (arrow) and left thalamus (arrow head) relative to the corresponding regions in the left hemisphere.

In the individual analysis, 21 (58.3%) of 36 VLBW infants exhibited abnormal AI_voi_ values. Fifteen infants (41.7%) exhibited right > left asymmetry for cerebral glucose metabolism (Table 3). These asymmetries were observed in the central region, frontal lobes, temporal lobes, occipital lobe, caudate nucleus, putamen, and thalamus. Fig 3 includes a representative F-18 FDG brain PET image of a VLBW infant with right > left asymmetry. Six infants (16.7%) exhibited left > right asymmetry. These asymmetries were observed in the central region, frontal lobe, temporal lobe, parietal lobe, caudate nucleus, putamen, and thalamus. In the regional analysis, right > left asymmetry was observed more frequently and was more extensive than left > right asymmetry. The AI_voi_ values of all brain regions were significantly higher than 0 (*P* < 0.001).

**Table 3:** The number of very low-birth-weight infants exhibiting asymmetric cerebral glucose metabolism.

	Asymmetry index (n)		
	Left > Right	Normal	Right > Left
VLBW infants*	6	15	15
Region			
Central region	2	33	1
Lateral frontal lobe	0	35	1
Medial frontal lobe	1	31	4
Orbital frontal lobe	0	36	0
Lateral temporal lobe	0	35	1
Medial temporal lobe	1	33	2
Lateral parietal lobe	1	35	0
Lateral occipital lobe	0	34	2
Medial occipital lobe	0	36	0
Caudate nucleus	1	32	3
Putamen	2	33	1
Thalamus	2	25	9

*VLBW infant: vere-low-birth-weight infant.

**Fig 4:**
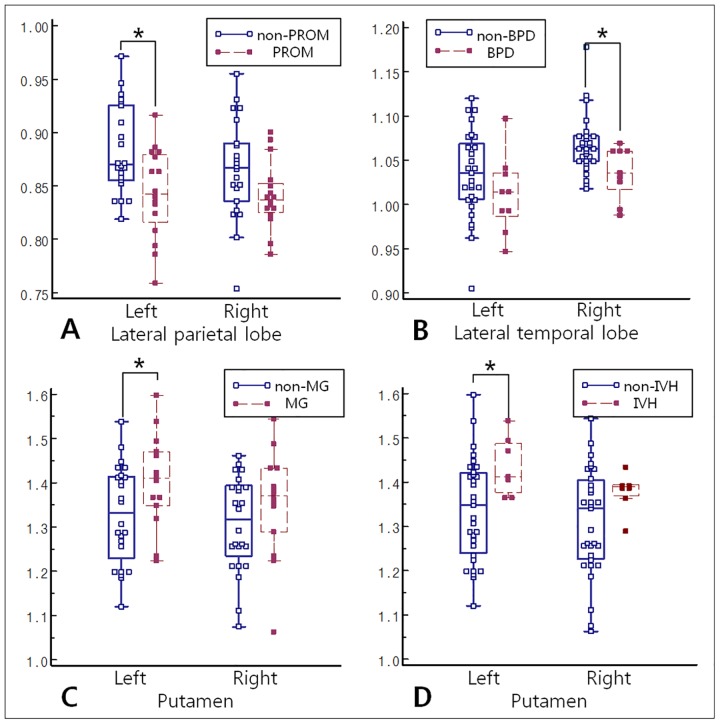
Comparison of CMRgl between subgroups. (A) The PROM group had a significantly lower CMRgl in the left parietal lobe than did the non-PROM group. (B) The BPD group had a significantly lower CMRgl in the right lateral temporal lobe than did the non-BPD group. (C, D) The MG and IVH groups had a significantly higher CMRgl in the left putamen than did the non-MG and non-IVH groups. PROM = premature rupture of membrane; CMRgl = cerebral glucose metabolic ratio; BPD = bronchopulmonary dysplasia; MG = multiple gestations, IVH = grade 1 or 2 intraventricular hemorrhage.

In the subgroup analyses, the PROM group had a significantly lower CMRgl in the left lateral parietal lobe than did the non-PROM group (*P* = 0.030) (Fig 4). The BPD group had a significantly lower CMRgl in the right central region and right lateral temporal lobe than did the non-BPD group (*P* = 0.038 and *P* = 0.035). The MG and IVH groups had a significantly lower CMRgl in the right medial occipital lobe than did the non-MG and non-IVH groups (*P* = 0.029 and *P* = 0.011, respectively). The MG and IVH groups had a significantly higher CMRgl in the left putamen than did the non-MG and non-IVH groups (*P* = 0.033 and *P* = 0.038, respectively). There were no significant differences in AI_voi_ between any of the subgroups. S1 and S2 Tables provide full results of the subgroup analyses.

## Discussion

In the present study, we demonstrated that more than half of the included VLBW infants exhibited asymmetric cerebral glucose metabolism, although there was no apparent evidence of parenchymal brain injury on brain MRI. Furthermore, the present study revealed that VLBW infants with neurodevelopmental risk factors had a significantly lower CMRgl in the left lateral parietal lobe than those without risk factors. Abnormalities in glucose metabolism that extend beyond the site of a structural lesion may help clinicians to determine the extent of injury in VLBW infants [11]. However, few studies have evaluated cerebral glucose metabolism in preterm infants, and these studies primarily included infants with severe brain injury [22] or a small number of low birth weight infants [13, 23]. The present study is the first to utilize F-18 FDG PET for the evaluation of cerebral glucose metabolism in a sufficient number of VLBW infants without structural abnormalities on brain MRI.

Cerebral glucose metabolism is usually homogeneous and symmetrical in adults with typical cognitive function/development. Generally, metabolic asymmetry is considered to be abnormal if it exceeds 10% [24]. Slight asymmetries of cerebral glucose metabolism have been observed in the Wernicke area, frontal eye fields, and angular gyrus, with a prevalence of generally less than 10% [20]. In accordance with the findings of previous studies, we observed that VLBW infants exhibited relatively high glucose metabolism in the thalamus, putamen, medial temporal lobe, and central region [13, 18]. However, in present study, 58.3% of VLBW infants exhibited abnormal AI_voi_ values (>10%), although there was no evidence of parenchymal brain injury on brain MRI. Thus, our findings suggest that microstructural white matter injury—which cannot be detected using MRI—disrupts corticostriatal connectivity and results in decreased activity of the cerebral cortex. This hypothesis is consistent with the known pathogenesis of macrostructural white matter injury in preterm infants, which can impair myelination of projection and association fibers or interfere with cortical neuronal development [25, 26].

Cerebral palsy develops in approximately 5 to 15% of surviving VLBW infants [5], while cognitive delay has been reported to occur in as many as 40% of these children upon reaching school age [3]. The characteristic symptoms of cerebral palsy are decreased or increased motor activity and abnormal posture, which can be symmetric or asymmetric [27]. The clinical phenotype of cerebral palsy can be predicted in VLBW infants based on patterns of cerebral glucose metabolism on F-18 FDG PET images. Kerrigan et al. reported that bilateral thalamic hypometabolism predicts the spastic diplegic type of cerebral palsy, and that the topography of focal cortical areas exhibiting hypometabolism is correlated with specific cognitive deficits [28]. Similar to the findings of previous studies, our results demonstrated that 41.7% of VLBW infants exhibited right dominant asymmetry, while 16.7% exhibited left dominant asymmetry. In addition, right dominant asymmetry was observed more frequently and was more extensive than left dominant asymmetry. Such findings may be explained by factors unique to the circulation of each fetus, as both prenatal factors and perinatal hypoxia-ischemia are known to play a major role in most cases of cerebral palsy [29]. In fetal circulation, the right common carotid artery is located closer to the left ventricle than the left common carotid artery and has two sources of blood flow from the left ventricle and ductus arteriosus [30]. A relatively weak blood supply from the left common carotid artery can easily cause microstructural white matter injuries in prenatal and perinatal hypoxic states [31]. The unique cerebrovascular anatomy and physiology of premature infants underlies the exquisite sensitivity of the white matter to the abnormal milieu of preterm extrauterine life, particularly with regard to ischemia and inflammation [32].

Even in the absence of structural brain abnormalities, VLBW infants remain at high risk for cognitive dysfunction, impaired language development, and neurobehavioral impairment [5, 10]. Previously identified risk factors for poor cognitive development in VLBW infants include chorioamnionitis, MG, PROM, BPD, and IVH [5, 33, 34]. Many studies have revealed that clinical risk factors can reliably predict neurodevelopmental impairment in VLBW infants [33–35]. Lodha et al. [35] reported that the Clinical Risk Index for Babies score predicted major neurodevelopmental impairment (odds ratio, 1.57; 95% CI, 1.26–3.01) and poor outcome (odds ratio, 1.46; 95% CI, 1.31–1.71) at 36 months’ corrected age. In agreement with previous studies, our results revealed that glucose metabolism in the cerebral cortices of VLBW infants with PROM, BPD, MG or IVH were significantly lower than those in infants without risk factors. The decreased glucose metabolism was shown in the lateral parietal and temporal lobes, the medial occipital lobes and central region depending on the risk factors. This result provides indirect evidence that F-18 FDG PET can detect microstructural abnormalities, which are related with poor neurodevelopmental outcome, in VLBW infants.

The present study possesses some limitation of note. First, we did not perform VOI analysis based on partial volume correction. The partial volume effect can influence the quantitative analysis of PET images, resulting in a lower CMRgl than the actual value. However, we believe that the partial volume effect on the AI_voi_ was minimal in the present study: Bilateral cerebral hemispheres without structural abnormalities on MRI were analyzed using the same method, and the partial volume effect on CMRgl was similar in both cerebral hemispheres. Second, we did not directly investigate the association between long-term neurodevelopmental outcomes and cerebral glucose metabolism in VLBW infants. Although the present study revealed asymmetry of cerebral glucose metabolism, further longitudinal studies are required to relate F-18 FDG PET images to long-term neurodevelopmental outcomes. Finally, the radiation exposure to the infants during F-18 FDG PET may raise concerns if proposed for clinical use in the prediction of neurodevelopmental outcomes. However, most PET scans in neonates can be accomplished using effective doses approximately equal to the yearly background radiation exposure, or less than the exposure during a clinical CT scan of the head [36, 37].

## Conclusion

Our findings demonstrated that VLBW infants without structural abnormalities have asymmetry of cerebral glucose metabolism. Decreased cerebral glucose metabolism are noted in infants with neurodevelopmental risk factors. F-18 FDG PET could show microstructural abnormalities not detected by MRI in VLBW infants.

## Supporting information

S1 Table Comparison of metabolic ratios between PROM and non-PROM groups and between BPD and non-BPD groups. (DOCX)

S2 Table Comparison of metabolic ratios between MG and non-MG groups and between IVH and non-IVH groups. (DOCX)

S1 Data Data underlying the findings. (XLSX)
