# Supplementary material for: Asymmetry of cerebral glucose metabolism in very low-birth-weight infants without structural abnormalities
Source: PLoS One. 2017 Nov 2;12(11):e0186976. doi: 10.1371/journal.pone.0186976 (PMC5667759; doi:10.1371/journal.pone.0186976)
Supplement: S1 Table — (DOCX) [file pone.0186976.s001.docx]

**S1 Table. Comparison of metabolic ratios between PROM and non-PROM groups and between BPD and non-BPD groups**

| VOI | Side | PROM^†^ | | | BPD^‡^ | | |
| --- | --- | --- | --- | --- | --- | --- | --- |
|  |  | Y | N | *P* | Y | N | *P* |
| Central region | Right | 1.06 ± 0.04 | 1.07 ± 0.04 | 0.437 | 1.04 ± 0.03 | 1.07 ± 0.04 | 0.038^*^ |
|  | Left | 1.05 ± 0.05 | 1.07 ± 0.05 | 0.424 | 1.05 ± 0.05 | 1.06 ± 0.05 | 0.645 |
| Lateral frontal | Right | 0.89 ± 0.04 | 0.91 ± 0.05 | 0.201 | 0.92 ± 0.05 | 0.89 ± 0.04 | 0.077 |
|  | Left | 0.9 ± 0.04 | 0.91 ± 0.04 | 0.356 | 0.94 ± 0.04 | 0.89 ± 0.03 | 0.009^a^ |
| Medial frontal | Right | 0.92 ± 0.06 | 0.92 ± 0.05 | 0.867 | 0.93 ± 0.06 | 0.92 ± 0.05 | 0.703 |
|  | Left | 0.89 ± 0.05 | 0.88 ± 0.05 | 0.625 | 0.91 ± 0.05 | 0.88 ± 0.05 | 0.200 |
| Orbital frontal | Right | 0.92 ± 0.04 | 0.90 ± 0.04 | 0.071 | 0.90 ± 0.05 | 0.91 ± 0.04 | 0.624 |
|  | Left | 0.9 ± 0.04 | 0.88 ± 0.04 | 0.124 | 0.89 ± 0.06 | 0.89 ± 0.03 | 0.896 |
| Lateral temporal | Right | 1.07 ± 0.04 | 1.06 ± 0.03 | 0.180 | 1.04 ± 0.03 | 1.07 ± 0.03 | 0.035^*^ |
|  | Left | 1.03 ± 0.05 | 1.03 ± 0.04 | 0.863 | 1.02 ± 0.04 | 1.04 ± 0.05 | 0.346 |
| Medial temporal | Right | 1.22 ± 0.12 | 1.19 ± 0.12 | 0.492 | 1.14 ± 0.14 | 1.22 ± 0.12 | 0.137 |
|  | Left | 1.23 ± 0.11 | 1.16 ± 0.12 | 0.083 | 1.14 ± 0.13 | 1.2 ± 0.12 | 0.264 |
| Lateral parietal | Right | 0.85 ± 0.03 | 0.87 ± 0.05 | 0.145 | 0.85 ± 0.03 | 0.86 ± 0.05 | 0.820 |
|  | Left | 0.85 ± 0.03 | 0.88 ± 0.04 | 0.030 ^*^ | 0.88 ± 0.03 | 0.87 ± 0.04 | 0.740 |
| Lateral occipital | Right | 0.88 ± 0.04 | 0.9 ± 0.04 | 0.470 | 0.88 ± 0.04 | 0.89 ± 0.04 | 0.374 |
|  | Left | 0.85 ± 0.04 | 0.87 ± 0.05 | 0.277 | 0.87 ± 0.03 | 0.86 ± 0.05 | 0.894 |
| Medial occipital | Right | 0.98 ± 0.03 | 0.97 ± 0.03 | 0.482 | 0.98 ± 0.04 | 0.98 ± 0.03 | 0.737 |
|  | Left | 0.97 ± 0.04 | 0.96 ± 0.04 | 0.671 | 0.97 ± 0.03 | 0.96 ± 0.04 | 0.464 |
| Caudate nucleus | Right | 0.89 ± 0.07 | 0.90 ± 0.10 | 0.616 | 0.93 ± 0.10 | 0.89 ± 0.08 | 0.208 |
|  | Left | 0.88 ± 0.06 | 0.90 ± 0.10 | 0.552 | 0.93 ± 0.09 | 0.88 ± 0.08 | 0.166 |
| Putamen | Right | 1.29 ± 0.13 | 1.34 ± 0.10 | 0.176 | 1.32 ± 0.08 | 1.32 ± 0.12 | 0.911 |
|  | Left | 1.33 ± 0.12 | 1.37 ± 0.11 | 0.299 | 1.37 ± 0.08 | 1.35 ± 0.13 | 0.646 |
| Thalamus | Right | 1.36 ± 0.20 | 1.46 ± 0.15 | 0.098 | 1.43 ± 0.18 | 1.42 ± 0.18 | 0.929 |
|  | Left | 1.29 ± 0.18 | 1.39 ± 0.20 | 0.158 | 1.34 ± 0.19 | 1.35 ± 0.20 | 0.862 |

^*^Statistically significant results; ^†^PROM = premature rupture of membrane; ^‡^BPD = bronchopulmonary dysplasia
