# Supplementary material for: Asymmetry of cerebral glucose metabolism in very low-birth-weight infants without structural abnormalities
Source: PLoS One. 2017 Nov 2;12(11):e0186976. doi: 10.1371/journal.pone.0186976 (PMC5667759; doi:10.1371/journal.pone.0186976)
Supplement: S2 Table — (DOCX) [file pone.0186976.s002.docx]

**S2 table. Comparison of metabolic ratios between MG and non-MG groups and between IVH and non-IVH groups**

| VOI | Side |  | MG^†^ |  | IVH^‡^ | | |
| --- | --- | --- | --- | --- | --- | --- | --- |
|  |  | Y | N | *P* | Y | N | *P* |
| Central region | Right | 1.06 ± 0.02 | 1.07 ± 0.04 | 0.411 | 1.06 ± 0.03 | 1.07 ± 0.04 | 0.795 |
|  | Left | 1.07 ± 0.05 | 1.06 ± 0.05 | 0.732 | 1.08 ± 0.05 | 1.06 ± 0.05 | 0.244 |
| Lateral frontal | Right | 0.9 ± 0.04 | 0.89 ± 0.05 | 0.570 | 0.91 ± 0.05 | 0.9 ± 0.05 | 0.576 |
|  | Left | 0.91 ± 0.04 | 0.9 ± 0.04 | 0.342 | 0.91 ± 0.04 | 0.90 ± 0.04 | 0.659 |
| Medial frontal | Right | 0.90 ± 0.06 | 0.93 ± 0.05 | 0.079 | 0.91 ± 0.05 | 0.93 ± 0.06 | 0.473 |
|  | Left | 0.89 ± 0.05 | 0.89 ± 0.05 | 0.933 | 0.89 ± 0.06 | 0.89 ± 0.05 | 0.953 |
| Orbital frontal | Right | 0.91 ± 0.05 | 0.91 ± 0.04 | 0.869 | 0.92 ± 0.03 | 0.9 ± 0.04 | 0.423 |
|  | Left | 0.9 ± 0.05 | 0.88 ± 0.03 | 0.384 | 0.9 ± 0.04 | 0.89 ± 0.04 | 0.408 |
| Lateral temporal | Right | 1.07 ± 0.04 | 1.06 ± 0.02 | 0.161 | 1.06 ± 0.02 | 1.06 ± 0.03 | 0.954 |
|  | Left | 1.04 ± 0.06 | 1.03 ± 0.04 | 0.694 | 1.04 ± 0.08 | 1.03 ± 0.04 | 0.510 |
| Medial temporal | Right | 1.22 ± 0.15 | 1.19 ± 0.11 | 0.467 | 1.15 ± 0.09 | 1.21 ± 0.13 | 0.215 |
|  | Left | 1.17 ± 0.13 | 1.20 ± 0.12 | 0.569 | 1.13 ± 0.06 | 1.20 ± 0.13 | 0.180 |
| Lateral parietal | Right | 0.85 ± 0.04 | 0.86 ± 0.04 | 0.617 | 0.86 ± 0.03 | 0.86 ± 0.04 | 0.921 |
|  | Left | 0.86 ± 0.05 | 0.88 ± 0.04 | 0.156 | 0.87 ± 0.04 | 0.87 ± 0.04 | 0.665 |
| Lateral occipital | Right | 0.89 ± 0.04 | 0.89 ± 0.04 | 0.631 | 0.88 ± 0.04 | 0.89 ± 0.04 | 0.438 |
|  | Left | 0.86 ± 0.04 | 0.87 ± 0.05 | 0.587 | 0.88 ± 0.04 | 0.86 ± 0.05 | 0.385 |
| Medial occipital | Right | 0.96 ± 0.03 | 0.99 ± 0.03 | 0.029 ^*^ | 0.95 ± 0.04 | 0.98 ± 0.03 | 0.011^*^ |
|  | Left | 0.95 ± 0.03 | 0.97 ± 0.04 | 0.106 | 0.96 ± 0.03 | 0.96 ± 0.04 | 0.855 |
| Caudate nucleus | Right | 0.9 ± 0.10 | 0.90 ± 0.08 | 0.989 | 0.89 ± 0.08 | 0.90 ± 0.09 | 0.698 |
|  | Left | 0.91 ± 0.10 | 0.88 ± 0.08 | 0.298 | 0.92 ± 0.08 | 0.88 ± 0.09 | 0.243 |
| Putamen | Right | 1.35 ± 0.13 | 1.30 ± 0.10 | 0.204 | 1.38 ± 0.04 | 1.31 ± 0.12 | 0.137 |
|  | Left | 1.41 ± 0.11 | 1.32 ± 0.11 | 0.033^*^ | 1.44 ± 0.07 | 1.33 ± 0.12 | 0.038^*^ |
| Thalamus | Right | 1.43 ± 0.18 | 1.41 ± 0.18 | 0.732 | 1.47 ± 0.14 | 1.41 ± 0.18 | 0.399 |
|  | Left | 1.39 ± 0.22 | 1.33 ± 0.18 | 0.403 | 1.40 ± 0.21 | 1.34 ± 0.19 | 0.444 |

^*^Statistically significant results; ^†^MG = multiple gestation; ^‡^IVH = intraventricular hemorrhage
